# Identification of citrullinated peptides in the synovial fluid of patients with rheumatoid arthritis using LC-MALDI-TOF/TOF

**DOI:** 10.1007/s10067-016-3247-4

**Published:** 2016-04-08

**Authors:** Fei Wang, Fang-Fang Chen, Wen-Bo Gao, Hai-Yong Wang, Ning-Wei Zhao, Min Xu, De-Yu Gao, Wei Yu, Xiao-Ling Yan, Jian-Ning Zhao, Xiao-Jun Li

**Affiliations:** 1 Institute of Clinical Laboratory Science, Jinling Hospital, School of Medicine, Nanjing University, 305 East Zhongshan Road, Nanjing, 210002 China; 2 Department of Osteology, Jinling Hospital, School of Medicine, Nanjing University, 305 East Zhongshan Road, Nanjing, 210002 China; 3 Biomedical Research Laboratory, Shimadzu (China) Co., Ltd., Shanghai, 200052 China; 4 State Key Laboratory of Analytical Chemistry for Life Science, Department of Chemistry, Nanjing University, Nanjing, 210093 China

**Keywords:** Citrullinated protein, LC-MALDI-TOF/TOF, Rheumatoid arthritis, Synovial fluid

## Abstract

The objective of the study is to investigate potential citrullinated autoantigens as targets of anti-citrullinated protein antibodies (ACPAs) response in synovial fluids (SFs) of patients with rheumatoid arthritis (RA). SFs from six RA patients and six osteoarthritis (OA) patients as controls were collected. The citrullinated proteins in SFs were extracted by immunoprecipitation with rabbit anti-citrulline antibodies. Matrix-assisted laser desorption/ionization time of flight mass spectrometry/time of flight mass spectrometry (MALDI-TOF/TOF) mass spectrometry was subsequently performed to discover a characteristic neutral loss to finally determine citrullinated autoantigens. A total of 182 citrullinated peptides and 200 citrullinated sites were identified in RA SFs, while 3 citrullinated peptides and 4 citrullinated sites were identified in OA SFs. The 182 citrullinated peptides from RA SFs and the 3 citrullinated peptides from OA SFs were derived from 83 and 3 autoantigens, respectively. Eighty-three autoantigens except protein-arginine deiminase type-2 (PADI2) and protein-arginine deiminase type-2 (PADI4) were over-citrullinated compared with controls, and the citrullinated sites of PADI2 and PADI4 were different in two groups. Interestingly, citrullinated histone H3.3 (H3F3A) was found in OA controls, but not in RA groups. The differential citrullinated proteins identified in RA SFs suggested potential autoantigens were targeted for ACPAs response and might contribute to the induction and perpetuation of complement activation and joint inflammation in RA.

**Electronic supplementary material:**

The online version of this article (doi:10.1007/s10067-016-3247-4) contains supplementary material, which is available to authorized users.

## Introduction

Rheumatoid arthritis (RA) is an autoimmune disease characterized by the formation of inflammatory, invasive tissue, and rheumatoid pannus in synovial membranes, subsequently resulting in joint destruction and systemic complications. The related autoimmunity is often associated with certain major histocompatibility complex (MHC) types and the presence of anti-citrullinated protein antibodies (ACPAs) [1]. ACPAs are important biomarkers of RA and can be detected even before the clinical onset of the disease; consequently, they are recognized as a predictive and diagnostic marker. Furthermore, ACPAs in the inflammatory synovium bind to citrullinated autoantigens to form immune complexes (ICs), which lead to the development of inflammation [2–7]. Thus, a simple and effective method is needed to detect citrullinated proteins in the joint fluid from RA patients.

**Fig. 1:**
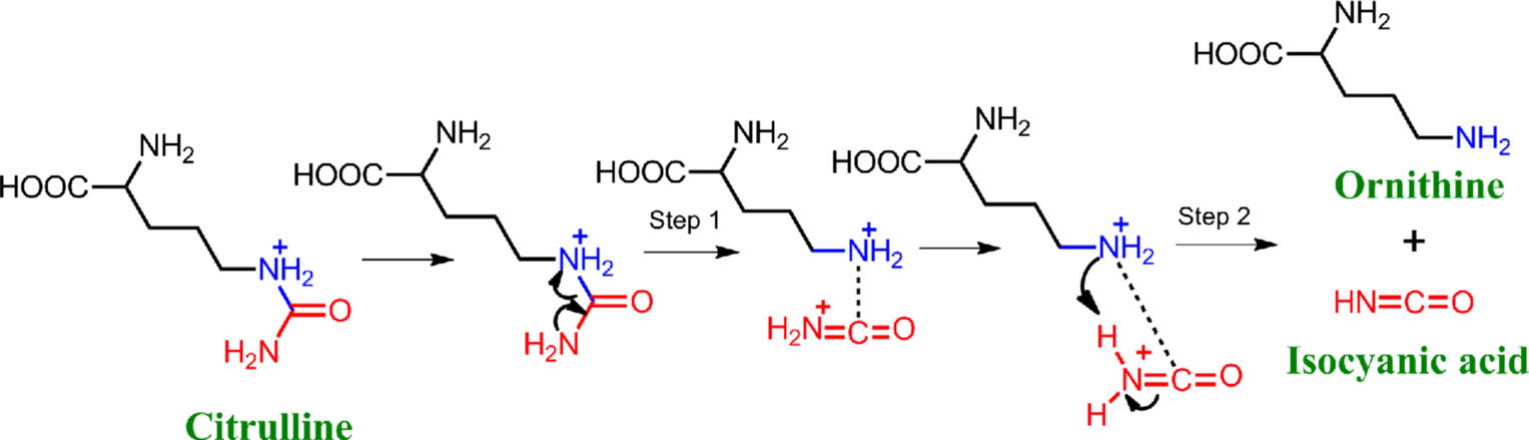
Schematic of the citrullinated peptide detection method. Step 1: citrullination of the protein. Step 2: the hydrogen bond is cleaved during CID of the citrullinated peptides, resulting in a signature 43-Da neutral loss from peptidyl-citrulline

Citrullination is a post-translational modification (PTM) involving the conversion of an arginine residue to a non-coded citrulline residue, catalyzed by peptidylarginine deiminases (PADIs). This PTM leads to the loss of a positive charge and a reduction in hydrogen-bonding ability [8]. The traditional method to detect citrullinated proteins in biological fluids is two-dimensional polyacrylamide gel electrophoresis (2D-PAGE) followed by immunoblotting and Fourier transform ion cyclotron resonance (ICR) mass spectrometry (MS) analysis, which is labor-intensive and time-consuming [9–12]. Moreover, the mass shift of citrullination is very small (+1 Da), which can result in false positives [4,13]. Hao et al. [14] found that one specific signature of citrullination is that the neutral loss of 43 Da from the peptidyl-citrulline can be observed after collision-induced dissociation (CID) during triple quadruple/linear ion trap (Q-Trap) mass spectrometry, which indicates the elimination of isocyanic acid from the citrulline ureido group as shown in (Fig. 1). However, this technology has not been applied to human fluid because the complexity of the protein mixture made detection of low-abundance proteins very difficult. In addition, the low mass resolution of Q-trap MS prevented the reliable peptide and PTM characterization as in the high mass resolution of time-of-flight (TOF) MS. Thus, we first applied liquid chromatography-matrix-assisted laser desorption/ionization (LC-MALDI)-TOF/TOF to detect citrullinated proteins in human RA synovial fluid (SF).

In this study, citrullinated proteins were extracted by immunoprecipitation using agarose-conjugated rabbit anti-citrulline antibodies, followed by LC-MALDI-TOF/TOF MS analysis. The peptide sequences and citrullinated sites in RA SFs or osteoarthritis (OA) SFs were identified. The high-energy CID mode of MALDI-TOF/TOF (20 keV collision energy) was adopted to improve fragmentation efficiency for reliable peptide and PTM characterization, thereby enabling the identification of potential autoantigens for ACPAs.

## Methods

### Human sample collection

Samples of SFs were consecutively obtained from knee joints of patients with RA or patients with OA, as a control, during knee therapeutic arthrocentesis at the Department Osteology, Jinling Hospital, School of Medicine, Nanjing University from January 2011 to January 2012. In order to improve the possibility of the existence of citrullinated antigens, patients of RA fulfilling the criteria (serum ACPA >200 U/ml and synovial fluids ACPA >200 U/ml) were selected and SFs of patients who were diagnosed as OA were collected. Patients who had incomplete data were excluded. The diagnosis of RA was made according to the American College of Rheumatology/European League Against Rheumatism Collaborative Initiative 2010 criteria [15] and the diagnosis of OA was based on the 1986 clinical and radiological criteria for the diagnosis of knee OA developed by the American College of Rheumatology [16]. Finally, six patients with RA were obtained and six patients with OA were selected by random. The patients’ basic information and serologic profile are shown in (Table 1). Informed consent was obtained from all subjects and the study was approved by the local ethics committee (Nanjing, China).

**Table 1:** Clinical and laboratory characteristics of the RA and OA patients

Diagnosis	Sex	Age (years)	ACPA, (U/ml)	RF (IU/ml)	ESR (mm/h)	CRP (mg/dl)	Disease duration (years)	DAS28
RA	Female	61	>200	86.3	64	25.1	3	4.28
RA	Female	79	>200	397	109	42.7	4	4.93
RA	Female	73	>200	40.6	44	37.5	2	3.84
RA	Male	69	>200	24.5	29	27.3	3	3.43
RA	Male	63	>200	142	44	77.8	5	3.72
RA	Male	70	>200	102	82	31.4	2	4.16
OA	Female	68	<0.5	<20	8	8.9	2	_
OA	Female	53	<0.5	<20	7	7.8	3	_
OA	Female	63	<0.5	<20	5	2.5	2	_
OA	Male	69	<0.5	<20	3	0.6	5	_
OA	Male	54	<0.5	<20	7	4.3	4	_
OA	Male	58	<0.5	<20	14	9.9	3	_

*ACPA* anticitrullinated protein antibody, *RF* rheumatoid factor, *ESR* erythrocyte sedimentation rate, *CRP* C-reactive protein, *DAS28* disease activity score at 28 joints

### Immunoprecipitation

All SF samples were centrifuged at 25,000×*g* for 10 min at 4 °C and the supernatants were stored separately in sterile conditions at −80 °C. EDTA was added at a final concentration of 50 mM, followed by centrifugation at 14,000×*g* for 10 min at 4 °C, and the supernatant or sample was transferred to a new vial. Protein concentrations were determined by BioSpec-nano (Shimadzu Biotech, Kyoto, Japan), and 1 mg was then subjected to immunoprecipitation [17,18]. Anti-citrulline polyclonal antibody (pAb; Abcam, Cambridge, USA) was cross-linked to protein G-Agarose (Sigma Aldrich, St Louis, MO, USA) with stable amide linkages according to the manufacturer’s instructions. Citrullinated proteins were immunoprecipitated by incubating the supernatant/sample with agarose-conjugated rabbit anti-citrulline antibodies overnight. The samples were washed three times with 50 mM ammonium acetate pH 7.4 and once with H_2_O, and the bound peptides were then eluted with acetonitrile/H_2_O (7:3 *v*/*v*) containing 5 mM HCl. The eluted peptides were subjected to desalting by C18 tip (SciLifeLab, Shanghai, China). The efficiency of immunoprecipitation was determined on equal amounts of protein/sample (OA, RA, washes of RA, and eluents of RA) by immunoblotting using anti-citrulline pAb.

### Protein reduction, alkylation, and enzyme digestion

An additional 937 μL of 50 mM NH_4_HCO_3_ was added to the eluted proteins. The proteins were reduced by adding 10 μL of 0.5 M dithiothreitol (DTT) in 50 mM NH_4_HCO_3_ to a final concentration of 4 mM and incubated for 20 min at 56 °C. For alkylation, 27 μL of 0.55 M iodoacetamide was added, and the samples were incubated for 15 min at room temperature in the dark. The final concentration of the extracted proteins was approximately 2.4 mg/mL (total volume approximately 1250 μL) according to the optical density at 280 nm. ProteaseMax solution (10 μL of 1 %) was added together with 50 μL of 1 μg/μL LysC before incubation in a hydrated chamber at 37 °C overnight. The reaction was quenched by adding formic acid (FA; Fluka, Sigma) to a final concentration of 0.5 %. The digested peptides were then subjected to desalting by C18 tip (Shimadzu Biotech, Kyoto, Japan). Finally, the desalted peptides were immediately applied to a prominence nano2D-HPLC and Accuspot™ system (Shimadzu Biotech, Kyoto, Japan).

### 2D-NanoLC fractionation

A 5-μL aliquot of the desalted peptide/sample was loaded directly onto a PolySulfoethyl A Column (1.0 mm × 50 mm, 5 μm) for the first dimensional strong cation exchange separation (*A* = 10 mM FA, *B* = 600 mM FA). The samples were then subjected to a second dimensional RP separation using a Capillary EX-Nano MonoCap C-18 column (0.16 mm × 150 mm, 5 μm): *A* = 5 % acetonitrile + 0.1 % FA, *B* = 95 % acetonitrile + 0.1 % FA. The flow rate of the system was set at 4 μL/min post-split. The eluent passed through a UV/Vis detector (220 nm) and was mixed with α-cyano 4-hydroxycinnamic acid matrix (CHCA; 5 mg/ml in 50/50 acetonitrile/0.1 % trifluoroacetic acid) and deposited onto a stainless steel MALDI target using the Accuspot™ LC-MALDI deposition robot [19].

### LC-MALDI-TOF/TOF MS analysis

Samples were deposited at a volume of ∼1 μL/spot. The signal-to-noise ratio (S/N) was determined using Launchpad version 2.9.1 software (Shimadzu Biotech, Kyoto, Japan). The limit of detection (LOD) was established with a S/N of 3:1. The m/z value was calibrated with 50 fmol each of human angiotensin II and human adrenocorticotropin fragment 18–39 and 250 fmol each of bovine insulin oxidized beta chain and bovine insulin as external standards. The m/z reported in MALDI-TOF/TOF (MALDI-7090, Shimadzu Kratos, Manchester, UK) was set in positive ion mode and a mass range of 1000–4000 Da. The peptide ions with high S/N (S/N > 10:1) were subjected to CID for subsequent MS/MS analysis.

### Bioinformatics analysis

The raw MS/MS data were searched using the Mascot engine and then processed with PTM Finder™ Software (Shimadzu Kratos) using the following criteria: database, Swiss-Prot, human; enzyme, LysC; miscleavages, 2; static modifications, carbamidomethylation of cysteine (+57.02 Da); variable modifications, oxidation of methionine (+16.00 Da); neutral loss of isocyanic acid from peptidyl-citrulline (−43.02 Da); precursor ion tolerance, 0.3 Da; fragment ion tolerance, 0.8 Da. At this point, the citrullinated sites were identified by MS/MS analysis of the AA(8)AA ion because an ornithine residue (Orn) was expected to be the product after loss of a carbamyl group. Thus, the neutral loss of isocyanic acid from peptidyl-citrulline could be differentiated from the deamidation of peptidyl-asparagine or peptidyl-glutamine. All entries were filtered using a false positive rate of 1 % at the peptide levels, and false positives were removed. The citrullinated proteins from the RA SFs were further analyzed with DAVID Bioinformatics Resources (David 6.7 software, Bethesda, Maryland, USA) to understand their biological functions.

## Results

A total of 182 citrullinated peptides and 200 citrullinated sites were identified in the RA SFs, while only three citrullinated peptides and four citrullinated sites were identified in the OA SFs (Tables 2 and 3). The 182 citrullinated peptides from the RA SFs were derived from 83 autoantigens, and the three citrullinated peptides from the OA SFs were derived from three autoantigens. The autoantigens in the RA SFs were over-citrullinated compared with the controls. Among these, 26 citrullinated proteins identified here have also been validated in previous studies (Table 4), which suggests that this strategy for identifying citrullinated peptides is highly effective.

**Table 2:** Citrullinated peptides and their deaminized sites identified by MALDI-TOF-MS in the RA SFs

Gene name	Protein ID	Peptide sequence	Citrullinated sites
A2M	A2MG_HUMAN	DNGCF**R**SSGSLLNNAIK	R1081
		GN**R**IAQWQSFQLEGGLK	R174
		EQAPHCICANG**R**QTVSWAVTPK	R853
		FQVDNNN**R**LLLQQVSLPELPGEYSMK	R1297
ACTG1	ACTG_HUMAN	DLYANTVLSGGTTMYPGIAD**R**MQK	R312
		AGFAGDDAP**R**AVFPSIVGRPRHQGVMVGMGQK	R28
ALB	ALBU_HUMAN	AWAVARLSQ**R**FPK	R246
		LCTVATL**R**ETYGEMADCCAK	R105
		VHTECCHGDLLECADD**R**ADLAK	R281
		**R**MPCAEDYLSVVLNQLCVLHEK	R469
		YLYEIAR**R**HPYFYAPELLFFAK	R169
		CCTESLVNR**R**PCFSALEVDETYVPK	R509
ANXA1	ANXA1_HUMAN	DITSDTSGDF**R**NALLSLAK	R177
		GTDVNVFNTILTT**R**SYPQLRRVFQK	R228
APOA1	APOA1_HUMAN	ENGGA**R**LAEYHAK	R212
		VEPLRAELQEGA**R**QK	R155
		DSG**R**DYVSQFEGSALGK	R51
		PALEDL**R**QGLLPVLESFK	R239
APOB	APOB_HUMAN	LEGTT**R**LTRK	R3386
		LTTNG**R**FREHNAK	R1689
		AEFTG**R**HDAHLNGK	R3020
		GNVATEISTE**R**DLGQCDRFK	R207
		IREVTQ**R**LNGEIQALELPQK	R2449
		**R**LIDLSIQNYHTFLIYITELLK	R4519
		YTYNYEAESSSGVPGTADSRSAT**R**INCK	R75
ARHGAP4	F5GZW3_HUMAN	EEQEVSWTQYTQ**R**K	R486
		AE**R**FSS**R**GGRLGSSREHQSFRK	R73, R77
		ELLGKTSV**R**QGLGPASTTSPSPGPRSPK	R889
		LREAERQEEKRAG**R**SVPTTTAGATEAGPLRK	R198
ARPC1B	ARC1B_HUMAN	QSSQ**R**GLTA**R**ERFQNLDK	R294, R299
		PTLVILRINRAARCV**R**WAPNENK	R100
C1R	C1R_HUMAN	GFLAYYQAVDLDECAS**R**SK	R149
		MQTRAGS**R**ESEQGVYTCTAQGIWK	R420
		DCGQPRNLPNGDF**R**YTTTMGVNTYK	R388
C1S	C1S_HUMAN	AA**R**LPVAPLRK	R586
C2	CO2_HUMAN	SSGQWQTPGAT**R**SLSK	R77
C3	CO3_HUMAN	**R**RHQQTVTIPPK	R880
		VLLDGVQNP**R**AEDLVGK	R315
		TVAV**R**TLDPERLGREGVQK	R945
		GYTQQLAF**R**QPSSAFAAFVK	R1060
		ITH**R**IHWESASLLRSEETK	R1310
		PDGVFQEDAPVIHQEMIGGL**R**NNNEK	R1134
C4B	CO4B_HUMAN	ISA**R**FSDGLESNSSTQFEVK	R218
		VDFTLSSE**R**DFALLSLQVPLK	R80
		AAANQMRNFLVRASC**R**LRLEPGK	R1675
		SHALQLNN**R**QIRGLEEELQFSLGSK	R1349
C4BPA	C4BPA_HUMAN	PELVNG**R**LSVDK	R493
		NL**R**WTPYQGCEALCCPEPK	R353
C6	CO6_HUMAN	FRCDSG**R**CIARK	R150
		**R**SENINHNSAFK	R289
		SS**R**TSNPYRVPANLENVGFEVQTAEDDLK	R225
C9	CO9_HUMAN	NF**R**TEHYEEQIEAFK	R213
CAT	CATA_HUMAN	NAIHTFVQSGSHLAA**R**EK	R522
CD44	CD44_HUMAN	NG**R**YSISRTEAADLCK	R41
		EQWFGN**R**WHEGY**R**QTPK	R407, R413
CFH	CFAH_HUMAN	**R**ITC**R**NGQWSEPPK	R1149, R1153
		HGGLYHENM**R**RPYFPVAVGK	R340
		RGYRLSS**R**SHTL**R**TTCWDGK	R1210, R1215
		AQTTVTCMENGWSPTP**R**CIRVK	R441
		IPCSQPPQIEHGTINSS**R**SSQESYAHGTK	R885
		CNMGYEYSE**R**GDAVCTESGWRPLPSCEEK	R246
CFHR2	FHR2_HUMAN	SHSF**R**AMCQNGK	R254
CFI	CFAI_HUMAN	DNE**R**VFSLQWGEVK	R480
		TH**R**YQIWTTVVDWIHPDLK	R389
CHMP2A	CHM2A_HUMAN	MDLLFGR**R**K	R8
		DLVRTR**R**YVRK	R71
CLC	LEG10_HUMAN	YQVMVNGQSSYTFDH**R**IK	R115
CP	CERU_HUMAN	ALYLQYTDETF**R**TTIEK	R81
		NLAS**R**PYTFHSHGITYYK	R115
		ENLTAPGSDSAVFFEQGTT**R**IGGSYK	R415
		DNEDFQESN**R**MYSVNGYTFGSLPGLSMCAEDRVK	R258
		NMAT**R**PYSIHAHGVQTESSTVTPTLPGETLTYVWK	R830
CPB2	CBPB2_HUMAN	DHEELSLVASEAV**R**AIEK	R342
CTLA4	CTLA4_HUMAN	AMHVAQPAVVLASS**R**GIASFVCEYASPGK	R51
		AQLNLAT**R**TWPCTLLFFLLFIPVFCK	R18
ENO1	ENOA_HUMAN	TGAPCRSE**R**LAK	R403
		LAQANGWGVMVSH**R**SGETEDTFIADLVVGLCTGQIK	R372
F2	THRB_HUMAN	YTACETA**R**TP**R**DK	R94, R97
		DST**R**IRITDNMFCAGYK	R541
FF3	AFF3_HUMAN	DFLTD**R**SNQSHLVGVPK	R111
		EAAANGGSGP**R**APVGSINARTTSDIAK	R745
		YTSEDLTSSS**R**PNGNSLFTSASSSK	R926
		SPPAAVAVAVSAAAPPPAVPCAPAENAPAPAR**R**SAGK	R606
FGA	FIBA_HUMAN	NV**R**AQLVDMK	R160
		GLIDEVNQDFTN**R**INK	R84
FGB	FIBB_HUMAN	REEAPSL**R**PAPPPISGGGY**R**ARPAK	R60, R72
		EDGGGWWYNRCHAANPNG**R**YYWGGQYTWDMAK	R445
FGG	FIBG_HUMAN	YEASILTHDSSI**R**YLQEIYNSNNQK	R134
		YTGNTY**R**VGDTYERPK	R106
		WLPSSSPVTGY**R**VTTTPK	R1573
		DNRGNLLQCICTGNG**R**GEWK	R265
GC	VTDB_HUMAN	HLSLLTTLSN**R**VCSQYAAYGEK	R218
		HQPQEFPTYVEPTNDEICEAF**R**K	R149
		**R**SDFASNCCSINSPPLYCDSEIDAELK	R445
H1FX	H1X_HUMAN	VPWFDQQNG**R**TYLK	R86
		YSQLVVETI**R**RLGE**R**NGSSLAK	R57, R62
H2AFY	H2AY_HUMAN	SIAFPSIGSG**R**NGFPK	R318
HABP2	HABP2_HUMAN	EEFHEQSF**R**VEK	R391
		FCEIGSDDCYVGDGYSY**R**GK	R203
		LIANTLCNS**R**QLYDHMIDDSMICAGNLQK	R480
HIST2H2AC	H2A2C_HUMAN	TRIIPRHLQLAI**R**NDEELNK	R89
		GNYAE**R**VGAGAPVYMAAVLEYLTAEILELAGNAA**R**DNK	R43, R72
HMGB2	HMGB2_HUMAN	MSSYAFFVQTC**R**EEHK	R24
HNRNPA1L2	RA1L2_HUMAN	GGNFGG**R**SSGPYGGGGQYFAK	R284
HP	HPT_HUMAN	VSVNE**R**VMPICLPSK	R261
		YVMLPVADQDQCI**R**HYEGSTVPEK	R311
HPR	HPTR_HUMAN	VLVNE**R**VMPICLPSK	R203
HPX	HEMO_HUMAN	NFPSPVDAAF**R**QGHNSVFLIK	R102
HSP90AA1	Q8TBA7_HUMAN	AQAL**R**DNSTMGYMAAK	R620
		HNDDEQYAWESSAGGSFTV**R**TDTGEPMGRGTK	R173
HSPA1A	HSP71_HUMAN	LLQDFFNG**R**DLNK	R357
		EIAEAYLGYPVTNAVITVPAYFNDSQ**R**QATK	R155
HSPA5	GRP78_HUMAN	SDIDEIVLVGGST**R**IPK	R368
		**R**LIGRTWNDPSVQQDIK	R98
Ig kappa chain V-II region RPMI 6410	KV206_HUMAN	VSN**R**DSGVPDRFSGSGSGTDFTLK	R79
Ig lambda chain V-II region NEI	LV202_HUMAN	**R**PSGVSN**R**FSGSK	R56, R63
Ig lambda chain V-II region NIG-84	LV211_HUMAN	LLIYDVNS**R**PSGISN**R**FSGSK	R56, R63
IGHA1	IGHA1_HUMAN	YLTWAS**R**QEPSQGTTTFAVTSILRVAAEDWK	R282
IGHG3	IGHG3_HUMAN	SCDTPPPCP**R**CPEPK	R128
		TPLGDTTHTCP**R**CPEPK	R113
ING4	ING4_HUMAN	WFCP**R**CSQERK	R241
ITIH2	ITIH2_HUMAN	RLSNENHGIAQ**R**IYGNQDTSSQLK	R475
		TILDDLRAEDHFSVIDFNQNI**R**TW**R**NDLISATK	R356, R359
KNG1	KNG1_HUMAN	ICVGCP**R**DIPTNSPELEETLTHTITK	R268
KRT33B	KT33B_HUMAN	ETMQFLND**R**LASYLEK	R66
LBR	LBR_HUMAN	EAR**R**EVEVK	R111
		PLTSF**R**QRK	R61
		SAR**R**SASASHQADIK	R96
		ELAV**R**TFEVTPIRAK	R195
		AP**R**NDLSPASSGNAVYDFFIGRELNPRIGTFDLK	R353
LCP1	PLSL_HUMAN	GDEEGVPAVVIDMSGL**R**EK	R316
		ALENDPDC**R**HVIPMNPNTNDLFNAVGDGIVLCK	R141
LGALS3BP	LG3BP_HUMAN	SGGSD**R**TIAYENK	R514
		SQLVYQSR**R**GPLVK	R436
LRG1	A2GL_HUMAN	ALGHLDLSGN**R**LRK	R175
		LQVLGKDLLLPQPDL**R**YLFLNGNK	R239
MAPRE1	MARE1_HUMAN	PLTSSSAAPQRPISTQ**R**TAAAPK	R168
MMP8	MMP8_HUMAN	FYQLPSNQYQST**R**K	R52
MNDA	MNDA_HUMAN	INQEEVGLAAPAPTA**R**NK	R119
NCF1	NCF1_HUMAN	SGQDVSQAQ**R**QIK	R292
		STATDITGPIILQTY**R**AIANYEK	R162
ORM1	A1AG1_HUMAN	EQLGEFYEALDCL**R**IPK	R167
PABPC1	PABP1_HUMAN	AVTEMNG**R**IVATK	R356
		PVRIMWSQ**R**DPSL**R**K	R89, R94
		ITGMLLEIDNSELLHMLESPESL**R**SK	R604
PADI2	PADI2_HUMAN	VGVFYVENPFFGQ**R**YIHILGR**R**K	R225, R233
PADI4	PADI4_HUMAN	GFRLLLASP**R**SCYK	R495
		TL**R**EHNSFVERCIDWNRELLK	R536
		PFGPVING**R**CCLEEK	R609
POFUT2	OFUT2_HUMAN	VFVATDAV**R**K	R337
		DFIWGHRQDVPSLEGAV**R**K	R315
PPIA	PPIA_HUMAN	EGMNIVEAMERFGS**R**NGK	R148
PRG4	PRG4_HUMAN	DQYYNIDVPSRTA**R**AITT**R**SGQTLSK	R1386, R1391
PRKCD	KPCD_HUMAN	QSM**R**SEDEAK	R132
		SP**R**DYSNFDQEFLNEK	R628
		IIGRCTGTAANS**R**DTIFQK	R216
PTPN22	PTN22_HUMAN	GP**R**NPPPTWNI	R499
		PAESVQSNNSSSFLNFGFAN**R**FSK	R491
SAA2	SAA2_HUMAN	RGPGGAWAAEVISNA**R**ENIQRLTG**R**GAEDSLADQAANK	R80, R89
SERPINA3	AACT_HUMAN	ADLSGITGA**R**NLAVSQVVHK	R350
SERPINC1	ANT3_HUMAN	LVSAN**R**LFGDK	R177
		IPEATN**R**RVWELSK	R78
		ANS**R**FATTFYQHLADSK	R89
SLC22A4	S22A4_HUMAN	VPLTTSLFFVGVLLGSFVSGQLSD**R**FGRK	R166
STAT4	STAT4_HUMAN	NVSTLSN**R**RFVLCGTNVK	R173
		SLQSSSVSE**R**QRNVEHKVAAIK	R139
		FHGNPMHVAVVISNCL**R**EERRILAAANMPVQGPLEK	R110
TAGLN2	TAGL2_HUMAN	MAN**R**GPAYGLSREVQQK	R4
TF	TRFE_HUMAN	CSTSSLLEACTF**R**RP	R696
		EGYYGYTGAF**R**CLVEK	R541
		AD**R**DQYELLCLDNT**R**K	R239, R251
TNC	TENA_HUMAN	NG**R**ENFYQNWK	R2033
		RVTTT**R**LDAPSQIEVK	R802
		VEAARNLTVPGSL**R**AVDIPGLK	R1127
		PDTEYEVSLISR**R**GDMSSNPAK	R878
		ETFTTGLDAP**R**NLRRVSQTDNSITLEWRNGK	R897
TNC	TENA_HUMAN	VPEITRTVSGNTVEYALTDLEPATEYTL**R**IFAEK	R1866
TNFAIP6	TSG6_HUMAN	NFLAG**R**FSHL	R273
VIM	VIME_HUMAN	MALDIEIATY**R**K	R401
		PDLTAAL**R**DV**R**QQYESVAAK	R270, R273
VPRPB	VPRBP_HUMAN	FISGTP**R**RK	R707
		SPFGSSF**R**TFNATDYK	R1334

Citrullinated residues are indicated with a bold **R**

**Table 3:** Citrullinated peptides and their deaminized sites identified by MALDI-TOF-MS in the OA SFs

Gene name	Protein ID	Peptide sequence	Citrullinated sites
H3F3A	H33_HUMAN	DIQLAR**R**IRGERA	R130
PADI2	PADI2_HUMAN	GFPVVLDSP**R**DGNLK	R373
PADI4	PADI4_HUMAN	TLPVVFDSP**R**N**R**GLK	R372, R374

Citrullinated residues are indicated with a bold **R**

**Table 4:** 26 citrullinated proteins in our study were validated in previous studies

Protein ID	References
Arginine deiminase type-4	[19]
Alpha-1-acid glycoprotein 1	[12]
Alpha-2-macroglobulin	[12]
Annexin A1	[12]
Apolipoprotein A-I	[12]
Apolipoprotein B-100	[12]
Ceruloplasmin	[12]
C4b-binding protein alpha chain	[12]
Complement C2	[12]
Complement C4-B	[12]
Complement factor H	[12]
Enolase	[12,20]
Fibrinogen	[20]
Fibronectin	[12]
Hemopexin	[12]
HSP90	[12]
Histone	[8,21]
Inter-alpha-trypsin inhibitor heavy chain H2	[12]
Myeloid cell nuclear differentiation antigen	[12]
Plastin-2	[12,22]
Proteoglycan 4	[12]
Serotransferrin	[12]
Serum albumin	[12]
Tenascin	[12]
Vitamin D-binding protein	[12]
Vimentin	[11]

**Fig. 2:**
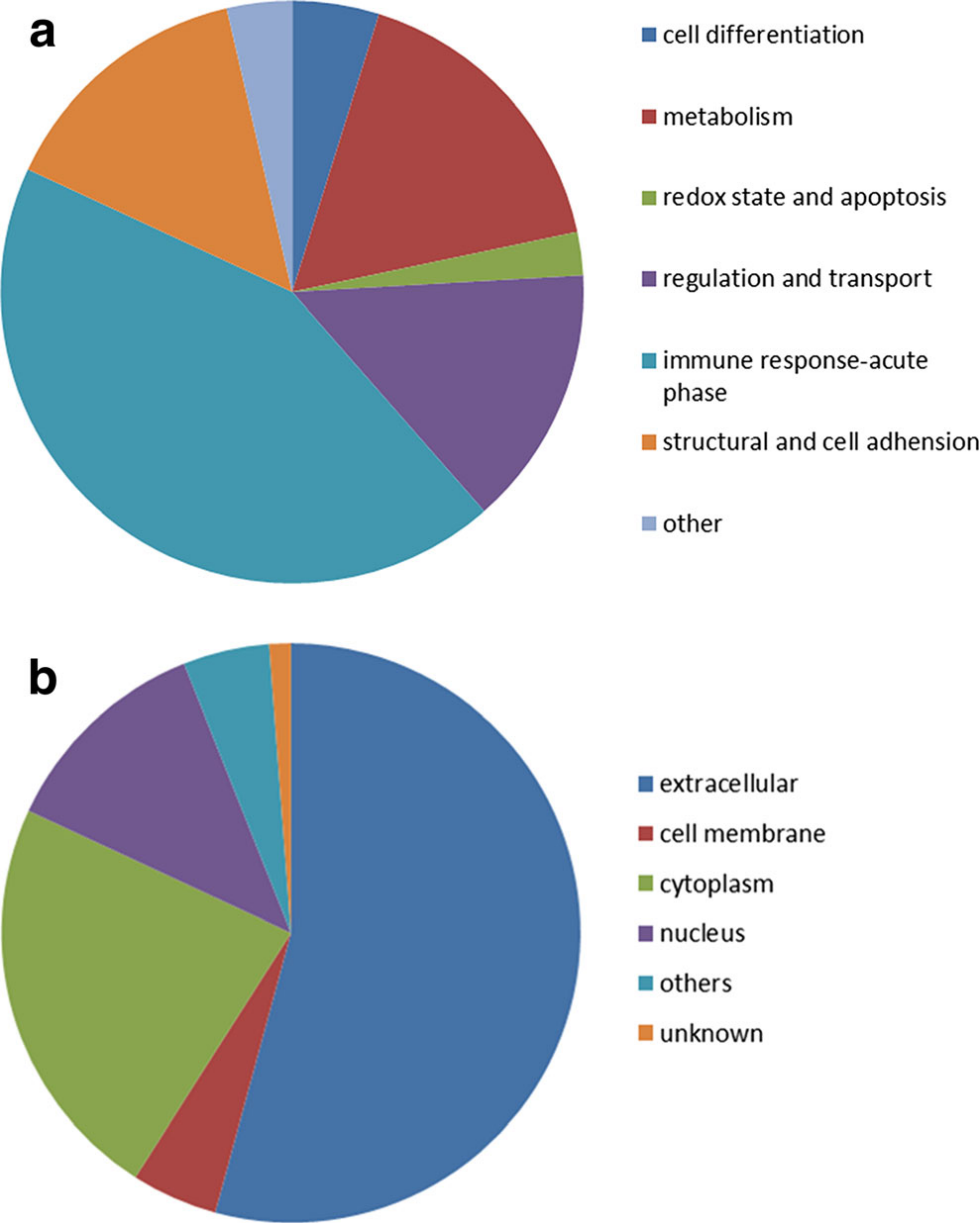
Classification by **a** function and **b** subcellular location of the proteins identified in the RA SFs

Functional analysis of the identified citrullinated proteins in the RA group was performed with David 6.7 software. The categories of “disease” and “gene ontology” reported a significant enrichment of RA and acute inflammatory response-associated genes, which corresponded to the physiological status of the patients in the present study (Supplement Fig. 1). Furthermore, genes involved in the enriched pathways in the list were associated with complement and coagulation cascades (Supplement Fig. 2). Additionally, proteins that were involved in cell differentiation, metabolism, redox state and apoptosis, regulation and transport, immune response and acute phase, structural and cell adhesion, and other groups based on the NCBI and UniProt database information are shown in (Fig. 2a). Moreover, the proteins were also classified by their subcellular location, as described in (Fig. 2b). The results of our analysis demonstrated that the citrullinated proteins obtained with our protocol provide reliable data on the state of citrullination in RA SF.

## Discussion

The major methods used currently to identify citrullinated proteins employ 2D-PAGE followed by immunoblotting and Fourier transform ion cyclotron resonance mass spectrometry analysis. For example, J.B.C. van Beers et al. found 192 proteins including 53 citrullinated proteins with their citrullinated residues in RA SFs [12]. One problem with this method is the small mass shift (+1 Da) from the conversion of peptidyl arginine to Cit, which is challenging for mass detection to distinguish. In the present study, citrullinated proteins were effectively enriched following immunoprecipitation (Supplement Fig. 1). NanoLC was then used to fractionate the tryptic digests of citrullinated proteins to improve the sensitivity and dynamic range of protein identification. With this method, not only are peptides of the same nominal mass isolated by temporal separation, but signal suppression is also reduced because of the separation of low- and high-abundance peptides. Importantly, the unique LC-MALDI peak picking algorithm promotes the MS/MS of selected ions at the apex of the eluting chromatographic peak to allow the most efficient data acquisition. This is not often the case with ES LC-MS/MS, where MS/MS acquisition is often taken on the rising edge of the eluting chromatographic peak. In addition, the high-energy CID mode of MALDI-TOF/TOF (20 keV collision energy, MALDI-7090) allowed us to determine the citrullinated sites more easily, according to the characteristic neutral loss of an isocyanic acid group from peptidyl-citrulline.

A number of chaperone molecules were identified within the SFs, particularly heat shock 70 kDa protein 1A/1B (HSPA1A), glucose-regulated protein 78 kDa (GRP78 or HSPA5), and HSP90AA1, members of the stress-inducible heat-shock protein 70 family. Also, we previously found GRP75 (HSP70) and binding immunoglobulin protein (BiP or GRP78) in RA synovial fibroblast-like synoviocytes (FLSs) [20]. Citrullinated BiP induces anti-CCP and anti-citrullinated fibrinogen antibodies and exacerbates collagen-induced arthritis in mice, and deaminated HSP90 was identified as a diagnostic autoantigen for a potentially serious manifestation of RA [10,21]. Recently, HSPs have been reported that not only act as chaperones during protein folding but also play a role between ubiquitin E3 ligase and the proteasome to inhibit proinflammatory NF-ΚB signaling [22]. In addition, both canonical and non-canonical NF-ΚBs are overexpressed in RA and are associated with the persistence of inflammation in RA [23]. Thus, citrullination of HSP may contribute to the chronic inflammation in the synovium or dysregulation of RA synovial fibroblasts, suggesting that citrullination may correlate with complement activation and the perpetuation of RA.

In a previous study from our group, we also reported that the elevated Annexin A11 in FLSs may be associated with the extensive synovial fibroblast-like synoviocytes hyperplasia. Additionally, in the extracellular environment defined as synovial fluid, we found citrullinated AnnexinA1, another member of the annexin superfamily of structurally related Ca^2+^-dependent phospholipid-binding proteins. Several other studies have demonstrated that AnnexinA1 is a glucocorticoid-induced molecule that can be transferred into cartilage and can modulate T cell function and the adaptive immune responses relevant to RA [24,25]. Consistent with this, treatment of mice with dexamethasone promotes potent antiarthritic effects that are dynamically attenuated in AnxA1^−/−^ mice [26]. Our observations on citrullinated Annexin A1 reflect the possibility that citrullinated or non-citrullinated Annexin may be a target to minimize glucocorticoid use in RA.

The different citrullinated sites of PADI2 and PADI4 in the two groups suggest new potential biomarkers for RA. PADI2 and PADI4 are the only PAD isotypes expressed in the synovial tissue of patients with RA, and they were reported to induce differentiation and apoptosis [27]. PADI4, found in the cell nucleus, mediated gene transcription by regulating arginine citrullination and methylation in histones H1, H3, and H4 and was autocitrullinated during cell activation [8,28,29]. Interestingly, citrullinated H3F3A was found in the OA controls, but not in the RA group. In addition, histones H1x and H2A were only citrullinated in the RA group. These results suggest that PADI2 and PADI4 represent a heterogeneous subtype with different citrullinated sites targeting multiple structural domains, where the specific citrullinated site may predict a specific disease. The exact mechanism underlying this phenomenon remains to be elucidated.

Although we identified the potential antigens for ACPA, some limitations remain, including the amount of patients was small, thus we pooled all samples per group to gain more sensitivity and to find more citrullinated antigens; results merely compared with previous studies; the validated process used only one method of mass spectrometry and was only on the basis of mass-spectrometry-based proteomics, so we performed DAVID Bioinformatics Resources to classify these genes corresponding to citrullinated proteins, at the same time, estimate and verify the reliability. Further studies will employ western blot to identify some selected potential autoantigens. At the same time, we will collect samples of synovial fluid or serum of RA patients as more as possible and then test antibodies corresponding to autoantigens in synovial fluid or serum of RA patients in order to obtain reliable results from clinical data. These limitations indicate the need for larger validation studies and prospective SFs studies in groups where larger samples are available.

Overall, we demonstrated a simple and efficient strategy for detecting citrullinated proteins and citrullinated sites in human RA SFs. In addition to the previously detected citrullinated proteins in RA SF, the novel citrullinated proteins identified by the data here may represent new antigens for ACPAs, as well as new markers for diagnosis. More importantly, this data will contribute to the search for the etiopathogenesis of, and new therapeutic targets for RA.

## Electronic supplementary material

Below is the link to the electronic supplementary material.

ESM 1 (DOCX 1136 kb)
