# Supplementary material for: Identification of citrullinated peptides in the synovial fluid of patients with rheumatoid arthritis using LC-MALDI-TOF/TOF
Source: Clin Rheumatol. 2016 Apr 8;35:2185–94. doi: 10.1007/s10067-016-3247-4 (PMC4989008; doi:10.1007/s10067-016-3247-4)
Supplement: Supplementary file 1 — (DOCX 1136 kb) [file 10067_2016_3247_MOESM1_ESM.docx]

**Identification of citrullinated peptides in synovial fluid of patients with rheumatoid arthritis using LC-MALDI-TOF/TOF**

**Fei Wang^#1^ · Fang-Fang Chen^#1^ · Wen-Bo Gao^1^ · Hai-Yong Wang^1^ · Ning-Wei Zhao^3^ · Min Xu^1^ · De-Yu Gao^1^ · Wei Yu^1^ · Xiao-Ling Yan^1^ · Jian-Ning Zhao^2^ · Xiao-Jun Li^1,4^**

#These two authors contributed equally to this work.

1 Institute of Clinical Laboratory Science, Jinling Hospital, School of Medicine, Nanjing University, 305 East Zhongshan Road, Nanjing 210002, China

2 Department of Osteology, Jinling Hospital, School of Medicine, Nanjing University, 305 East Zhongshan Road, Nanjing 210002, China

3 Biomedical Research Laboratory, Shimadzu (China) Co., Ltd. Shanghai 200052, China

4 State Key Laboratory of Analytical Chemistry for Life Science, Department of Chemistry, Nanjing University, Nanjing 210093, China

***Corresponding Author***

*****Xiao-Jun Li. Tel: +86(0)13805180607. Fax: +86 25 84803061. E-mail address: xiaojunli0725@163.com

***Contents***

1. **Fig. 1** The efficiency of immunoprecipitation
2. **Fig. 2** The functional analysis of citrullinated proteins


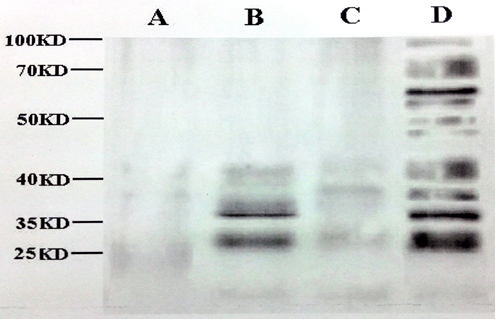


**Fig. 1** The efficiency of immunoprecipitation. A (OA SF samples), B (RA SF samples), C (washes of RA) and D (eluents of RA) were fractionated by sodium dodecyl sulfate-polyacrylamide gel electrophoresis and analyzed by immunoblotting using anti-citrulline pAb. The positions of molecular weight markers (KD) are indicated on the left.


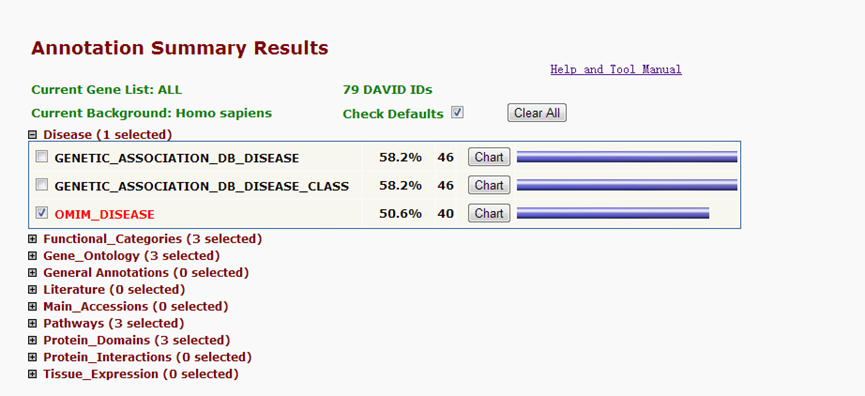


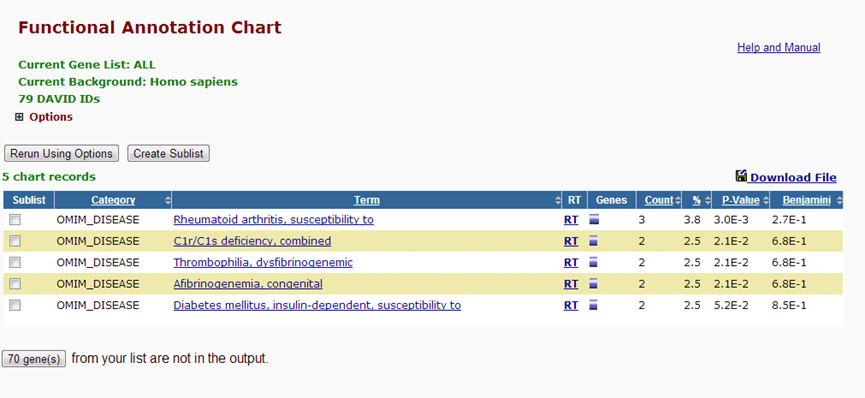


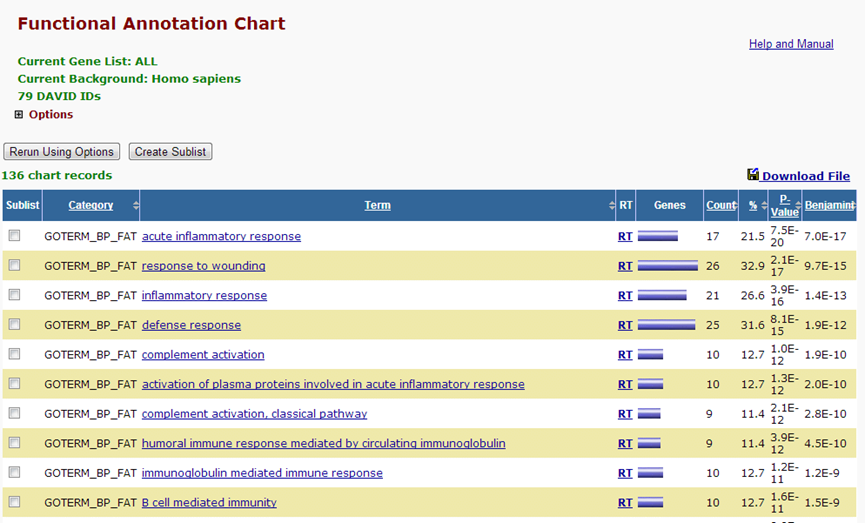


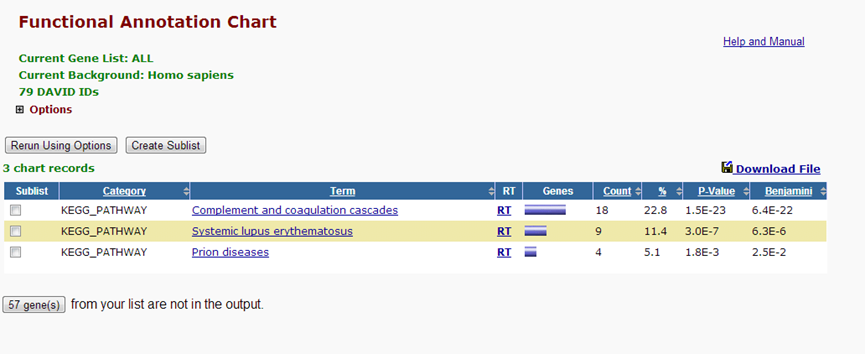


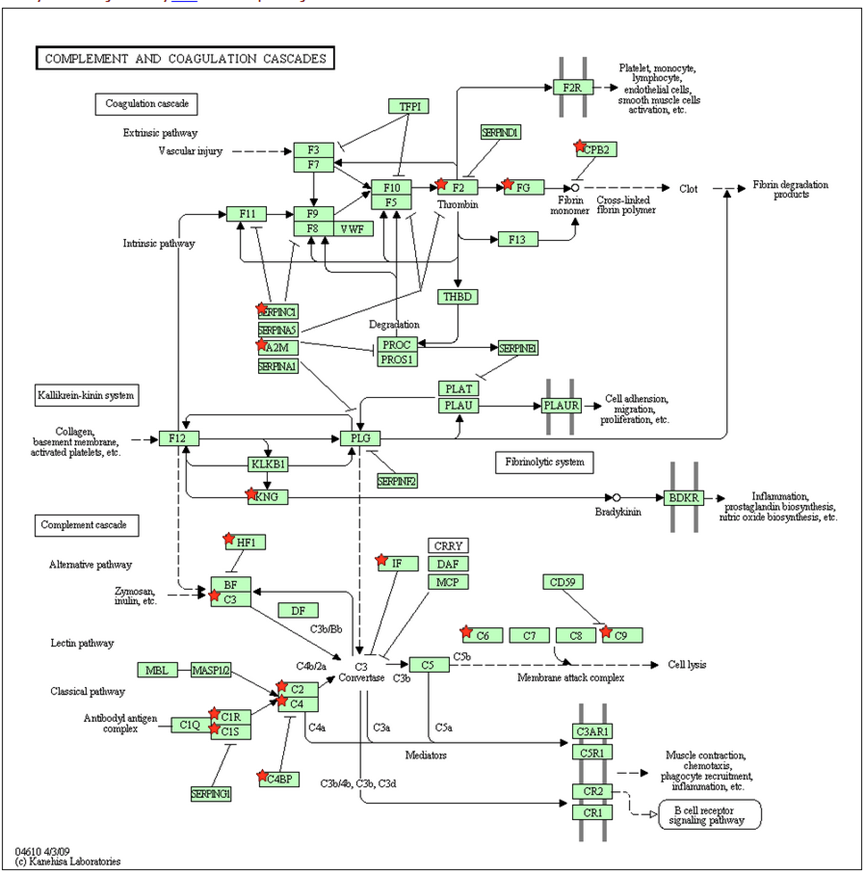


**Fig. 2** The functional analysis of citrullinated proteins by David 6.7 software
